# GatekeepR: an R Shiny application for the identification of nodes with high dynamic impact in Boolean networks

**DOI:** 10.1093/bioinformatics/btae007

**Published:** 2024-01-09

**Authors:** Felix M Weidner, Nensi Ikonomi, Silke D Werle, Julian D Schwab, Hans A Kestler

**Affiliations:** Institute of Medical Systems Biology, Ulm University, Albert-Einstein-Allee 11, Ulm 89081, Germany; Institute of Medical Systems Biology, Ulm University, Albert-Einstein-Allee 11, Ulm 89081, Germany; Institute of Medical Systems Biology, Ulm University, Albert-Einstein-Allee 11, Ulm 89081, Germany; Institute of Medical Systems Biology, Ulm University, Albert-Einstein-Allee 11, Ulm 89081, Germany; Institute of Medical Systems Biology, Ulm University, Albert-Einstein-Allee 11, Ulm 89081, Germany

## Abstract

**Motivation:**

Boolean networks can serve as straightforward models for understanding processes such as gene regulation, and employing logical rules. These rules can either be derived from existing literature or by data-driven approaches. However, in the context of large networks, the exhaustive search for intervention targets becomes challenging due to the exponential expansion of a Boolean network’s state space and the multitude of potential target candidates, along with their various combinations. Instead, we can employ the logical rules and resultant interaction graph as a means to identify targets of specific interest within larger-scale models. This approach not only facilitates the screening process but also serves as a preliminary filtering step, enabling the focused investigation of candidates that hold promise for more profound dynamic analysis. However, applying this method requires a working knowledge of R, thus restricting the range of potential users. We, therefore, aim to provide an application that makes this method accessible to a broader scientific community.

**Results:**

Here, we introduce *GatekeepR*, a graphical, web-based R Shiny application that enables scientists to screen Boolean network models for possible intervention targets whose perturbation is likely to have a large impact on the system’s dynamics. This application does not require a local installation or knowledge of R and provides the suggested targets along with additional network information and visualizations in an intuitive, easy-to-use manner. The Supplementary Material describes the underlying method for identifying these nodes along with an example application in a network modeling pancreatic cancer.

**Availability and implementation:**

https://www.github.com/sysbio-bioinf/GatekeepR https://abel.informatik.uni-ulm.de/shiny/GatekeepR/.

## 1 Introduction

We introduce an R Shiny application with a graphical user interface tailored to identify nodes exhibiting substantial impact on the dynamics of biological networks. To provide context, we will begin with a brief overview of Boolean network modeling. Furthermore, we will briefly summarize the derivation of the node classification and the significance of the threshold that used for the underlying approach (Weidner *et al.* 2021).

Boolean networks serve as mathematical representations of the dynamics within gene regulatory networks, initially conceptualized by Kauffman (1969). In these models, network components, or nodes, can represent the activity of genes, gene products such as mRNAs and proteins, or broader biological processes like apoptosis or the presence of stressors (Ikonomi *et al.* 2022). At any given moment *t*, the network’s state is denoted by a Boolean variable vector, x(t), belonging to the Boolean domain $B^{n}$. The evolution of the network’s state occurs by evaluating each node’s associated Boolean rule, connecting network components via logical operators such as AND, OR, and NOT (Schwab *et al.* 2020). Under the assumption of synchronous updates, where each node in the network is updated simultaneously during the transition from one point in time *t* to the next t+1, the dynamics eventually converge to stable states referred to as attractors. These attractors can be linked to distinct biological phenotypes (Kauffman 1993, Huang 1999).

Boolean networks have found successful applications in a wide array of biological systems and processes, including but not limited to cancer biology (Werle *et al.* 2022, 2023), stem cell biology (Ikonomi *et al.* 2020), developmental biology (Giacomantonio and Goodhill 2010), and the study of infectious diseases (Madrahimov *et al.* 2013). For a more comprehensive exploration of the theoretical underpinnings of Boolean networks (see e.g. Schwab *et al.* 2020).

One application of Boolean networks is the search for potential intervention targets, i.e. to perform *in silico* drug screening. However, this task can become complex in terms of runtime and memory. The dynamic state graph of Boolean networks grows exponentially with the increasing numbers of compounds in the system. On top of that, especially with the growing size of the network, the number of candidates to screen increases (Schwab and Kestler 2018). Therefore, an in-depth analysis of the changing dynamic behavior after perturbations of many different components or even combinations thereof can become infeasible.

Instead, the *GatekeepR* application aims to identify potential candidates for therapeutic targets based on the interaction graph of the networks. For larger networks, the interaction graph of a network with *n* nodes is orders of magnitude smaller than its corresponding state graph representing the networks’ dynamics ($2^{n}$ nodes). Our previous work (Weidner *et al.* 2021) has shown that relevant target candidates can be derived from the Boolean logic and graph-theoretical measures defined on the interaction graph, requiring no detailed analysis of the networks’ dynamics. This work can serve as a pre-selection of interesting candidates for a more detailed dynamic analysis. In fact, we identified a class of nodes called *gatekeepers* whose perturbations are likely to result in large changes in the dynamic attractor landscape while being rather lowly connected within the interaction graph. This class of nodes can be of special interest in drug-target screening, as it has the power to change the dynamics of a system significantly while being less central parts of the modeled system compared to e.g. so-called hubs. In the following, the rationale of the *gatekeepers* identification is briefly summarized.

In a first step, the combination of the measures of vertex betweenness (VB) (Freeman 1977) and determinative power (DP) (Heckel *et al.* 2013, Pentzien *et al.* 2018) was found to be the best-performing combination of predictors for a label classifying genes as having high or low dynamic impact. The measure of VB quantifies the number of shortest paths between any pairs of nodes which pass through a given network component. Nodes scoring high on VB thus act as bottlenecks in the network structure (Yu *et al.* 2007). The measure of DP is a sum of mutual information values calculated using a binary Shannon entropy (Pentzien *et al.* 2018). It uses a nodes’ regulatory rule to quantify the degree to which knowledge of a nodes’ state determines the state of all its outputs. Notably, scoring network components according to these measures requires no evaluation of network dynamics or the attractor landscape. The perturbation of network components by overexpression or knockout was taken to have a high dynamic impact if it affected the system’s attractor landscape. Dynamic changes were considered relevant when, upon perturbation, a large fraction of attractors of the unperturbed network were lost, while simultaneously a large number of new attractors were generated, which had a large minimal Hamming distance when compared to all previously present attractors. Thus, the attractor landscapes before and after the perturbation are as different as possible.

Based on these measures, a selection threshold *T* was determined, which excludes nodes ranking low on network measures of VB and DP.

That is, nodes are selected if they belong to the intersection $VB_{T}\cap DP_{T}$ where

(1) $$VB_{T}=\left\{g:r_{g}\leq \left\lceil Tn\right\rceil ,r=rk(VB)\right\}$$

for the case of VB for a network of *n* components where $r={\left(r_{g}\right)}_{g=1}^{n}$ and rk() is the ranking function (analogous definition for $DP_{T}$, see Weidner *et al.* 2021) for further details on the node selection and determination of an optimal threshold *T*).

While the selected nodes showed significantly higher dynamic impact than the non-selected components, this selection also included highly connected central regulators, called hub nodes. These hubs are defined by z-transformed connectivity $C_{i}\geq 2.5$, as given by Guimera and Nunes Amaral (2005). These z-scores are defined over the degree distribution of the interaction graph as

$$C_{i}=\frac{\delta_{i}-\bar{\delta }}{\sigma }.$$

where $\delta_{i}$ denotes the total number of in- and outgoing connections of network component $n_{i}$, $\bar{\delta }$ is the network’s mean degree, and σ its standard deviation.

While the selection confirmed the impact of perturbations of hubs on network dynamics, such nodes are not the preferred targets for interventions. This is due to the difficulty of reliably targeting such hubs (Dufour *et al.* 2013) and the possibility of unwanted side effects, in particular lethality (Jeong *et al.* 2001). Therefore, the selected nodes were further classified to individuate a class of nodes which also shows a high impact on dynamics while being more sparsely connected. This was done by calculating a mismatch between ranking on static network measures and rankings on connectivity. If this mismatch is positive, i.e. the node ranks higher on VB and DP than on connectivity, it is recommended as a target. All nodes in this class are further ranked by the magnitude of this mismatch. Since it was further found that these nodes preferentially relayed information to hub nodes, the class was given the name of *gatekeeper* nodes (Weidner *et al.* 2021).

Further details on this procedure are given in the Supplementary Material.

Perturbations of such nodes may then be investigated in more detail using tools such as e.g. ViSiBooL (Schwab *et al.* 2016) to obtain more extensive information on the activity patterns of attractors resulting from a specific perturbation.

## 2 Implementation

The *GatekeepR* application utilizes the R Shiny framework (Chang *et al.* 2017) to provide a graphical user interface to screen for drivers of the network’s dynamics. Thus, it is user-friendly and easy to interact with as no programming knowledge is required.

The application supports network models in the common SBML3: qual standard for qualitative models (Chaouiya *et al.* 2013) and a simple text-format as specified for the BoolNet R-package (Müssel *et al.* 2010).

As all calculations rely only on the Boolean functions and their resulting interaction graph rather than the evaluation of network dynamics, the list of recommended targets can be quickly obtained even for larger Boolean networks (BNs). For example, the analysis of a cancer network with 66 nodes and 211 interactions (Werle *et al.* 2023) required 12 s on a device with an Intel 2.4 GHz i9-12900 processor and 32 GB of RAM, while a network with 131 nodes and 313 interactions (Madrahimov *et al.* 2013) required 21 s.

A wide range of biological network models is available at the Cell Collective (Helikar *et al.* 2012), which can be downloaded as SBML files. Some of these models have been provided in the github repository associated with this work for exemplary analyses.

## 3 Features

**Figure 1.**
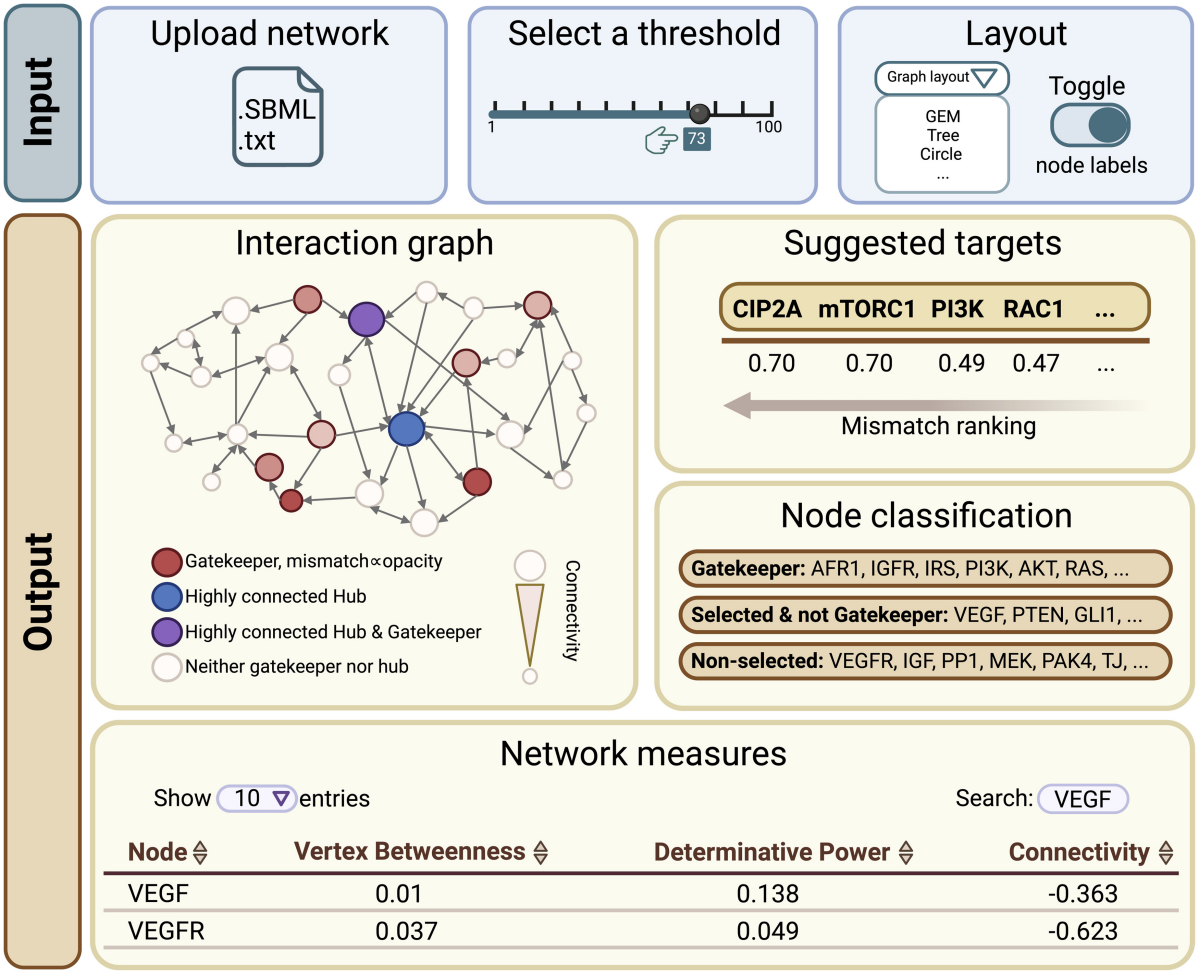
Overview of features of the GatekeepR R Shiny application. Users can upload networks containing Boolean rules either as text files formatted for processing in BoolNet or in SBML3: qual format. The selection threshold can be changed via a slider and is set to the default optimal value of T=73%. The classification will be automatically performed for the specified selection threshold, listing the recommended intervention targets ranked by the mismatch between their scores on network measures and connectivity. The network’s interaction graph is plotted with gatekeeper and hub nodes being highlighted in red and blue, respectively. Hubs which are also classified as gatekeepers are colored in purple. Higher mismatch values for gatekeepers are indicated by higher opacity. The size of nodes is proportional to their connectivity. The user can choose between various layouts for the graph and toggle the presence node labels. A table lists the values for all nodes on the measures of Vertex Betweenness, Determinative Power, and z-transformed connectivity. This table includes a sorting as well as search functionality.

Some of the features of the *GatekeepR* application include, see Fig. 1:

- Network information: The number of nodes and interactions in the network are listed along with the runtime for the performed analysis. The full classification of nodes into *gatekeepers*, selected but *non-gatekeeper* nodes and *low-impact non-selected* nodes is provided.
- Network interaction graph: Users can visualize the network interaction graph, with *gatekeeper* nodes as well as central hub regulators distinctly highlighted. The size of nodes is proportional to their connectivity. Higher ranks in the list of targets are indicated by higher opacity. The resulting graph can directly be saved as a PDF file.
- Adjustable selection threshold: Users can fine-tune the strictness of the selection threshold. This slider is set to the recommended value of T=73% by default, which has yielded balanced values for sensitivity and specificity in a binary classification of nodes into high and low dynamic impact classes. Changing this value will lead to a reclassification of nodes and may thus change the number and order of recommended targets.
- Layout variability: The application supports multiple layout structures commonly used in network visualization, including options specifically intended for plotting large graphs while avoiding overlapping nodes.

Figure 1 shows an overview of the features of the application, including user input options and the provided results. The user interface is further showcased in Supplementary Fig. S1. The specific targets listed here are results of the analysis of a 66-node network modeling pancreatic neuroendocrine tumors (PanNETs) (see Werle *et al.* 2023). The top ranked recommendations for this network are the nodes mTORC1 and CIP2A. Inhibition of the former is an approved treatment for PanNETs (Orr-Asman *et al.* 2017), while the latter has already been shown to be a relevant target in other cancer types (Wiegering *et al.* 2013, Soofiyani *et al.* 2017). This is elaborated further in the Supplementary Material.

The list shown under “Suggested intervention targets, sorted by mismatch” includes the recommended intervention targets. High mismatch values indicate a node ranking highly on VB and DP, which serves as an indicator of high dynamic impact, while also being sparsely connected. Such sparsely connected *gatekeepers* may be preferential targets since direct perturbations of central hub regulators could be lethal (Jeong *et al.* 2001). The presented order of nodes is thus intended to be read as a prioritization for further analyses and experiments.

To ensure interoperability with other network analysis tools, *GatekeepR* supports the analysis of networks in the commonly used SBML format, as it is used e.g. by the Cell Collective (Helikar *et al.* 2012). Furthermore, networks in this format can be imported into Cytoscape3 using the Cy3SBML plugin (König *et al.* 2012).

## Supplementary Material

btae007_Supplementary_Data
