# Supplementary material for: GatekeepR: an R Shiny application for the identification of nodes with high dynamic impact in Boolean networks
Source: Bioinformatics. 2024 Jan 9;40(1):btae007. doi: 10.1093/bioinformatics/btae007 (PMC11616774; doi:10.1093/bioinformatics/btae007)
Supplement: btae007_Supplementary_Data [file btae007_supplementary_data.pdf]

Supplementary information to:

GatekeepR: An R Shiny application for the identification of nodes with high dynamic impact in Boolean networks

1 Selection of network measures for capturing dynamic impact

The method implemented in the GatekeepR application aims to capture the impact of single-node perturbations (i.e. overexpressions or knockouts) on the attractor landscape without performing the dynamic analyses necessary to calculate the system's state transition graph (STG). Given the STG's exponential growth with network size, graph-based network measures are used instead which rely only on the evaluation of the system's logical rules and resulting interaction graph Weidner *et al.* (2021).

For this purpose a dataset of 35 published Boolean network models was analysed. These networks were checked to have a scale-free topology as is typical for biological networks using the poweRlaw R package (Gillespie (2014)).
These networks had a median size of $n = 18$ nodes with an interquartile range from 12.5 to 23. The smallest and largest networks contained $n = 5$ and $n = 51$ nodes respectively.
Given the size of these networks, their attractors could be calculated for the unperturbed systems as well as for all single-node overexpression and knockouts using the SAT-exhaustive attractor search in the BoolNet R package (Müssel *et al.* (2010); Dubrova and Teslenko (2011)).
A dynamic impact measure was defined, with nodes ranking highly if their perturbation causes the loss of many attractors existing in the unperturbed system while simultaneously leading to the appearance of many new attractors that show a different expression pattern from any previously existing attractors.
This dynamic impact was then used to generate a label that was to be predicted by network measures that do not rely on evaluation of dynamics.
Using various classification algorithms, it was found that the best performing models preferentially selected the measures of vertex betweenness (VB) and determinative power (DP) as shown in the supplementary material of Weidner *et al.* (2021).

The first of these measure, vertex betweenness, is defined for a vertex v (i.e. a network component or node) as

$$VB(v) = \sum_{v_i, v_j \in \mathcal{V} \setminus \{v\}} \frac{|s_{ij}(v)|}{|s_{ij}|} \tag{1}$$

and thus quantifies the ratio between the number of shortest paths s_{ij} connecting any pair of nodes i and j which passes through the node v. Nodes scoring highly on this measure can be interpreted as being located at bottlenecks in the network structure (Yu *et al.* (2007)).

The second measure, determinative powerHeckel *et al.* (2013); Pentzien *et al.* (2018), is an entropy-based measure which quantifies the reduction in uncertainty of the state of a nodes' outputs given knowledge of the state of the node itself. It is a sum over mutual information (MI) terms, which is itself defined via a binary Shannon entropy.
This entropy is defined as

$$h(p_g) = -p_g \log_2(p_g) - (1 - p_g) \log_2(1 - p_g) \tag{2}$$

where $p_g = P(X_g = 1)$ describes the probability of a random, binary variable X_g taking the value $x_g = 1$ (Matache and Matache (2016)).
To define the mutual information value, first the support of a Boolean function f_g is denoted as those states which are mapped to the output 1, that is

$$\mathcal{S}(f_g) = \{\mathbf{x} : f_g(\mathbf{x}) = 1\} \tag{3}$$

The mutual information is then given as

$$\begin{aligned} MI(X_g; f_{g'}(X)) = h\left(\sum_{\mathbf{x}\in\mathcal{S}(f_{g'})} p_{\mathbf{x}}\right) - \\ \sum_{b\in\{0,1\}} P(X_g = b)h\left(\sum_{\mathbf{x}\in\mathcal{S}(f_{g'})} P(X = \mathbf{x}|X_g = b)\right). \end{aligned} \tag{4}$$

where $p_{\mathbf{x}}$ is the probability of the state $\mathbf{x}$ and $X = (X_g)_{g=1}^n$. The determinative power is then given as a sum over these MI values as

$$DP_g = \sum_{g'=1}^{n} MI(X_g; f_{g'}(X)). \tag{5}$$

2 Determination of the optimal selection threshold

In the next step, an optimal selection threshold T was empirically determined by comparing the overlap of the top ranking $T\%$ of nodes according to the measures of VB and DP and comparing this selection to the top ranking $T\%$ according to the dynamic impact ranking (Weidner *et al.* (2021)).
Choosing for a balance between sensitivity and specificity in the selected and non-selected classes resulted in an optimal threshold value selecting those nodes in the top $T = 73\%$ in both VB as well as DP.

3 Identification of gatekeepers as a class of nodes

It was shown that the selected nodes included highly connected hub nodes whose perturbation had high dynamic impact. However, due to problems such as lethality of perturbations of such hubs, the selected nodes were further split to identify potential targets which retain high dynamic impact while being sparsely connected.
A comparison was thus made between the rankings of nodes in their respective networks according to the measures of VB and DP against their ranking on connectivity. These rankings were translated into percentile scores. Nodes who percentile scores were higher on VB and DP than on connectivity were designated positive mismatch (PM) nodes. In the opposite case, the selected nodes with higher connectivity than VB and DP scores were named negative mismatch (NM) nodes.
An investigation into mutual information flows from each of the three classes of nodes (PM, NM, non-selected) to hubs revealed paths along the interaction graph from PM nodes to hubs which carried a higher amount of mutual information than was the case for the other classes (Weidner *et al.* (2021)). Therefore, these nodes were named 'gatekeepers'.
Given the mismatch score, gatekeepers can be rank-ordered. Their identification requires only the calculation of VB and DP and thus does not rely on attractor evaluations.

4 Application: Gatekeepers as recommended targets in a PanNET model

Pancreatic neuroendocrine tumors (PanNETs) are a rare, slow-growing type of cancer whose underlying mechanisms are not yet fully understood.
Werle *et al.* (2023) have addressed this cancer type using a Boolean network model including $n = 66$ nodes. The model is therefore not amenable to exhaustive dynamic analysis using libraries such as BoolNet (Müssel *et al.* (2010)), and other approaches are required.
Using the GatekeepR method on this network returned two equally ranked top recommendations for the nodes mTORC1 and CIP2A.
In-silico simulations of such inhibition in the model of Werle *et al.* (2023) showed a reduction in the prevalence of states leading to pathological attractor states when compared to the unperturbed system. Notably, inhibition of mTORC1 is an already FDA-approved treatment for this type of cancer, e.g. using the drug Everolimus (Orr-Asman *et al.* (2017)).
The second top recommendation, CIP2A, has already been identified as a relevant factor in other cancer types such as colon cancer (Wiegering *et al.* (2013); Soofiyani *et al.* (2017)). The CIP2A node has also been identified as a tumor driver in simulations of Werle *et al.* (2023), fitting to the defining characteristic of gatekeepers as nodes whose perturbation has a high impact on dynamics.

5 Visualisation of the user interface

The following Figure S1 shows the user interface of the GatekeepR app, including all inputs the user can use. These include elements for the uploading of networks, changing the selection threshold and plotting of the interaction graph as well as the search and sort functions for the measures of VB, DP and connectivity.
In the interaction graph, gatekeepers are coloured in red, with higher mismatches (and thus higher rankings in the target suggestion) corresponding to higher opacity. Highly connected hubs are coloured blue and hubs which were also classified as gatekeepers are coloured purple. The graph and node classification shown here are using the PanNET network of Werle *et al.* (2023).

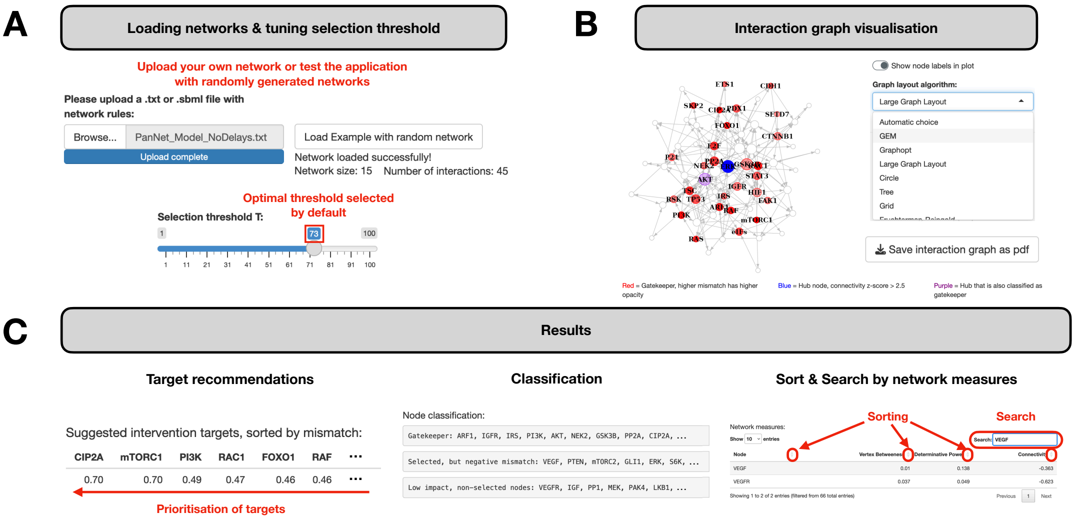

Figure S1: Screenshots of the user interface of the GatekeepR R Shiny application. (A) Users can upload networks as SBML files or text files formatted as required by BoolNet. Alternatively, results can be generated for randomly created networks to explore the application's features. (B) An interaction graph of the provided network is plotted, with gatekeepers and hubs being highlighted in colour. The user can choose a layout for plotting the graph and toggle the presence of node labels. The resulting plot can be saved as a PDF. (C) The analysis returns a list of recommended targets sorted by their mismatch score. A list of nodes in each class is provided as well as a table listing the values of VB, DP and connectivity for all nodes in the network. This table contains a sorting and search functionality.

References

Dubrova, E. and Teslenko, M. (2011). A SAT-Based Algorithm for Finding Attractors in Synchronous Boolean Networks. *IEEE/ACM Transactions on Computational Biology and Bioinformatics*, **8**(5), 1393–1399.

Gillespie, C. S. (2014). Fitting heavy tailed distributions: the poweRlaw package. *arXiv preprint arXiv:1407.3492*.

Heckel, R. *et al.* (2013). Harmonic analysis of Boolean networks: Determinative power and perturbations. *EURASIP Journal on Bioinformatics and Systems Biology*, **2013**(1), 6.

Matache, M. T. and Matache, V. (2016). Logical Reduction of Biological Networks to Their Most Determinative Components. *Bulletin of Mathematical Biology*, **78**(7), 1520–1545.

Müssel, C. *et al.* (2010). BoolNet—an R package for generation, reconstruction and analysis of Boolean networks. *Bioinformatics*, **26**(10), 1378–1380.

Orr-Asman, M. A. *et al.* (2017). mTOR Kinase Inhibition Effectively Decreases Progression of a Subset of Neuroendocrine Tumors that Progress on Rapalog Therapy and Delays Cardiac Impairment. *Molecular Cancer Therapeutics*, **16**(11), 2432–2441.

Pentzien, T. *et al.* (2018). Identification of biologically essential nodes via determinative power in logical models of cellular processes. *Frontiers in Physiology*, **9**, 1185.

Soofiyani, S. R. *et al.* (2017). The role of CIP2A in cancer: A review and update. *Biomedicine & Pharmacotherapy*, **96**, 626–633.

Weidner, F. M. *et al.* (2021). Capturing dynamic relevance in Boolean networks using graph theoretical measures. *Bioinformatics*, **37**(20), 3530–3537.

Werle, S. D. *et al.* (2023). A systems biology approach to define mechanisms, phenotypes, and drivers in PanNETs with a personalized perspective. *npj Systems Biology and Applications*, **9**(1), 22.

Wiegering, A. *et al.* (2013). CIP2A Influences Survival in Colon Cancer and Is Critical for Maintaining Myc Expression. *PLOS ONE*, **8**(10), e75292.

Yu, H. *et al.* (2007). The Importance of Bottlenecks in Protein Networks: Correlation with Gene Essentiality and Expression Dynamics. *PLOS Computational Biology*, **3**(4), e59.
